# Engineered Monoclonal Antibody with Novel Antigen-Sweeping Activity *In Vivo*

**DOI:** 10.1371/journal.pone.0063236

**Published:** 2013-05-07

**Authors:** Tomoyuki Igawa, Atsuhiko Maeda, Kenta Haraya, Tatsuhiko Tachibana, Yuki Iwayanagi, Futa Mimoto, Yoshinobu Higuchi, Shinya Ishii, Shigero Tamba, Naoka Hironiwa, Kozue Nagano, Tetsuya Wakabayashi, Hiroyuki Tsunoda, Kunihiro Hattori

**Affiliations:** Research Division, Chugai Pharmaceutical Co., Ltd., Gotemba, Shizuoka, Japan; Consejo Superior de Investigaciones Cientificas, Spain

## Abstract

Monoclonal antibodies are widely used to target disease-related antigens. However, because conventional antibody binds to the antigen but cannot eliminate the antigen from plasma, and rather increases the plasma antigen concentration by reducing the clearance of the antigen, some clinically important antigens are still difficult to target with monoclonal antibodies because of the huge dosages required. While conventional antibody can only bind to the antigen, some natural endocytic receptors not only bind to the ligands but also continuously eliminate them from plasma by pH-dependent dissociation of the ligands within the acidic endosome and subsequent receptor recycling to the cell surface. Here, we demonstrate that an engineered antibody, named sweeping antibody, having both pH-dependent antigen binding (to mimic the receptor-ligand interaction) and increased binding to cell surface neonatal Fc receptor (FcRn) at neutral pH (to mimic the cell-bound form of the receptor), selectively eliminated the antigen from plasma. With this novel antigen-sweeping activity, antibody without *in vitro* neutralizing activity exerted *in vivo* efficacy by directly eliminating the antigen from plasma. Moreover, conversion of conventional antibody with *in vitro* neutralizing activity into sweeping antibody further potentiated the *in vivo* efficacy. Depending on the binding affinity to FcRn at neutral pH, sweeping antibody reduced antigen concentration 50- to 1000-fold compared to conventional antibody. Thereby, sweeping antibody antagonized excess amounts of antigen in plasma against which conventional antibody was completely ineffective, and could afford marked reduction of dosage to a level that conventional antibody can never achieve. Thus, the novel mode of action of sweeping antibody provides potential advantages over conventional antibody and may allow access to the target antigens which were previously undruggable by conventional antibody.

## Introduction

Therapeutic monoclonal antibodies are now becoming an important option for treating various diseases [1], [2]. Although high affinity antibodies with neutralizing activity against various antigens have been generated and shown to be therapeutically effective *in vivo*, some clinically important antigens have proved difficult to target by conventional antibody because of the huge antibody dosage required.

It has been reported that administering conventional antibodies to target soluble antigens, such as amyloid beta [3], MCP1 [4], hepcidin [5], IL6 [6], CD23 [7] and VEGF [8], results in more than 1000-fold increased antigen concentration over the baseline due to the accumulation of antibody-antigen complex in plasma. Since the half-life of the IgG antibody is very much longer than that of the antigen, the binding of antigen to antibody results in an increase in the plasma antigen concentration by reducing the clearance of the antigen [9]. The extent of increase in antigen concentration is determined by the difference in clearance between antigen and antibody-antigen complex [10]. As a striking example, administration of high affinity antibody against hepcidin, which has very rapid clearance, resulted in approximately 5,000-fold increase in plasma hepcidin concentration, requiring a huge antibody dosage of 300 mg/kg weekly to neutralize the hepcidin, which is an unrealistic dosage for therapeutic development [5]. In other cases, the baseline plasma concentration of the antigen may itself be extremely high, as in that of complement factor C5, the target antigen of eculizumab, which is in the range of µg/mL, in contrast to most therapeutic antibodies in the pg/mL or ng/mL range. Because of such high C5 concentration, eculizumab requires huge antibody dosage for efficient C5 neutralization [11], which makes eculizumab one of the highest annual dosages of the approved therapeutic antibodies. In theory, even an antibody with infinite affinity would need to be at a concentration higher than that of the total antigen to neutralize that antigen *in vivo* [12]. Therefore, when targeting soluble antigens with rapid clearance or high baseline plasma concentration, even conventional antibody with infinite affinity requires huge antibody dosage to achieve therapeutic efficacy. This impedes not only the development of subcutaneous formulations, which are important for chronic disease, but also the commercial development itself, because of increased manufacturing cost.

Since these issues stem from the fact that conventional antibody can only bind to the antigen and accumulates the antigen in plasma, engineered antibody that enables active and selective elimination of the antigen from plasma could overcome these issues. In the natural system, several cell surface endocytic receptors, such as asialoglycoprotein receptor [13], low-density lipoprotein receptor [14] and epidermal growth factor receptor [15], deliver ligands to the lysosome to eliminate the ligands from plasma. These receptors bind to the ligands at the cell surface and internalize the ligands into the cell. Since these receptors bind to the ligands pH dependently, they release the ligands in the acidic endosome, and while the released ligands are transferred to lysosome and degraded, the free receptors rapidly recycle back to the cell surface for another round of ligand elimination from plasma. Such properties make these receptors ideal for “sweeping” the ligands from the plasma. In this study, we engineered monoclonal antibody to mimic the endocytic receptor-like property so that it can exert antigen-sweeping activity and eliminate the antigen from the plasma.

We have investigated engineered monoclonal antibody to exert novel antigen-sweeping activity by simultaneously engineering both pH-dependent antigen binding, to mimic the pH-dependent receptor-ligand binding of such endocytic receptors, and increased FcRn binding affinity at neutral pH, to mimic the cell-bound form of the receptors. We demonstrate that the conversion of conventional antibody into sweeping antibody, which directly eliminates the antigen from plasma, could afford huge reduction of the antibody dosage to a level that conventional antibody even with infinite affinity cannot achieve and may allow access to target antigens which have previously been undruggable by conventional antibody.

## Materials and Methods

### Ethics Statement

Animal studies were performed in accordance with the Guidelines for the Care and Use of Laboratory Animals at Chugai Pharmaceutical Co., Ltd. under the approval of the company’s Institutional Animal Care and Use Committee. The company is fully accredited by the Association for Assessment and Accreditation of Laboratory Animal Care International (http://www.aaalac.org).

### Generation of Anti-IL-6R Antibodies with Increased Binding Affinity to FcRn at Neutral pH

pH-dependent binding antibody against human soluble IL-6 receptor (hsIL-6R) with neutralizing activity (PH-IgG1) were generated from non-pH-dependent binding antibody (NPH-IgG1) as previously described [16]. pH-dependent binding antibodies against hsIL-6R without neutralizing activity (PHX-IgG1) in BaF/gp130 assay [17] were also generated. To increase the binding affinity to either mouse FcRn (mFcRn) or human FcRn (hFcRn) at neutral pH, various Fc-engineered variants were generated by site-directed mutagenesis of human IgG1. Amino acid substitutions were introduced at the positions 251–258, 286, 288, 307–316, 428 and 433–436 in the EU numbering system which were reported to affect FcRn binding [18]–[26]. Mutation was comprehensively introduced at each position, and effective mutations identified were combined to generate Fc variants with increased binding affinity to FcRn at neutral pH. More than 1,000 variants were generated and assessed for their binding affinity (K_D_) to recombinant mFcRn or hFcRn [18] at pH 7.0 using Biacore T200 (GE Healthcare). Each variant was captured onto a Protein L (ACTIgen) immobilized CM4 sensor chip, then FcRn was injected over the flow cell. K_D_ was determined using Biacore T200 Evaluation Software (GE Healthcare). Fc variants with the desired affinity to FcRn were identified. NPH-IgG1 (conventional antibody with neutralizing activity but without pH-dependent binding), PH-IgG1, PHX-IgG1 and their Fc variants were expressed transiently and purified. NPH-IgG1, PH-IgG1 and PHX-IgG1 were assessed for their K_D_ to recombinant hsIL-6R at pH 7.4 and pH 6.0 as previously described [16].

### *In vivo* Study of Antibodies in Normal Mice and hFcRn Transgenic Mice Co-injection Model

All animal experiments in this study were performed in accordance with the Guidelines for the Care and Use of Laboratory Animals at Chugai Pharmaceutical Co., Ltd. In co-injection model, C57BL/6J normal mice (Charles River) or hFcRn transgenic mice (hFcRn-Tgm, B6.mFcRn−/−.hFcRn Tg line 276+/+ mouse, Jackson Laboratories) [27] were administered by single i.v. injection with hsIL-6R alone or with hsIL-6R pre-mixed with antibody. The first group received 50 µg/kg hsIL-6R but the other groups additionally received 1 mg/kg of anti-IL-6R antibodies. Total hsIL-6R plasma concentration was determined as previously described [16].

### *In vivo* Study of Antibodies in a Normal Mice hsIL-6R Trans-signaling Model

To evaluate the effect of antibodies on hsIL-6R trans-signaling inhibition *in vivo*, C57BL/6J normal mice were initially i.v. injected with hsIL-6R [16] (250 µg/kg). Then antibodies with designated doses and MR16-1 [28] (15 mg/kg, rat anti-mouse IL-6R antibody) were administered at 2 h after the initial injection. 8 µg/kg of human IL-6 (Toray) was injected at 24 h. Blood samples were collected at 30 h after the initial injection and total hsIL-6R and serum amyloid A (SAA) plasma concentrations were determined as previously described [16].

### *In vivo* Study of Single Doses of Antibodies in Normal Mice and hFcRn Transgenic Mice Steady-state Model

An infusion pump (alzet) filled with 92.8 µg/mL hsIL-6R was implanted under the skin on the back of C57BL/6J normal mice or hFcRn-Tgm (B6.mFcRn−/−.hFcRn Tg line 32+/+ mouse, Jackson Laboratories) [27] to prepare model mice with constant plasma concentration of hsIL-6R. Monoclonal anti-mouse CD4 antibody GK1.5 [29] was administered by i.v. injection to inhibit the production of mouse antibody against hsIL-6R by depleting CD4+ T-cells. Antibodies against hsIL-6R were administered at 1 mg/kg to normal mice or hFcRn-Tgm with or without a single i.v. injection of 1 g/kg of hIgG (Intravenous immunoglobulin, CSL Behring) to mimic endogenous human IgG.

Plasma anti-hsIL-6R antibody concentration in the presence of human IgG was determined using anti-idiotype antibody coated on ELISA 96-well plates, and detected by hsIL-6R, biotinylated anti-hIL-6R antibody (R&D Systems) and Streptavidin-PolyHRP80 (Stereospecific Detection Technologies) using peroxidase substrate. Plasma total hsIL-6R, antibody concentration in the absence of hIgG and pharmacokinetic parameters were determined as previously described [16]. The theoretical free hsIL-6R concentration was calculated from antibody concentration, total hsIL-6R concentration and the K_D_ of the antibody by equilibrium reaction formula.

### *In vivo* Study of Multiple Doses of Antibodies in hFcRn Transgenic Mice Steady-state Model with High hsIL-6R Concentration

Study was performed as described in the single dose study but with 320 µg/mL hsIL-6R in the pump, and doses were administered to hFcRn-Tgm (B6.mFcRn−/−.hFcRn Tg line 32+/+ mouse, Jackson Laboratories) [27] every other day (except the first dose which was injected together with a single i.v. injection of 1 g/kg of human IgG). Total hsIL-6R plasma concentrations were determined as described above. To determine free hsIL-6R plasma concentration, samples were treated by rProtein A (GE healthcare) to remove antibody and antibody-antigen complex. Because rProtein A treatment requires 10 µL of plasma, samples of n = 3–5 were equally pooled before the treatment. Subsequently, the free hsIL-6R plasma concentrations were determined by the same method as for total hsIL-6R, and hsIL-6R neutralization percentages were obtained by calculating the percentage reduction of free hsIL-6R plasma concentration over control group.

### Pharmacokinetic Analysis and Simulation using Antibody-antigen Dynamic Model

The plasma concentration–time profiles of antibodies and total hsIL-6R obtained in the study of hFcRn-Tgm steady-state model were fitted to an antibody-antigen dynamic model [30] and parameters were optimized for conventional, pH-dependent binding, and v4-type sweeping antibodies. The k_a_ and k_d_ values were from surface plasmon resonance (SPR) data. Simulation study was carried out using the obtained pharmacokinetic parameters, and antibody dosages required to neutralize 95% of the antigen (baseline 250 ng/mL (6.6 nM)) at trough by dosing once a month were obtained for each type of antibody with antigen binding affinity (K_D_) of 0.001, 0.01, 0.1, 1 and 10 nM.

## Results

### Antigen Sweeping by pH-dependent Binding Antibody with Increased FcRn Binding at Neutral pH

**Figure 1:**
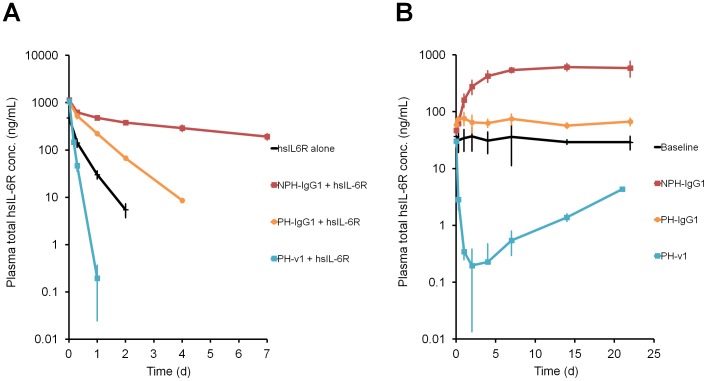
Antigen sweeping by pH-dependent antigen binding antibody with increased FcRn binding at neutral pH. *In vivo* study of NPH-IgG1, PH-IgG1 and PH-v1 in normal mice. Effect of antibodies on the total hsIL-6R plasma concentration was evaluated in a co-injection model and a steady-state model. In the co-injection model, hsIL-6R, hsIL-6R+NPH-IgG1, hsIL-6R+PH-IgG1 and hsIL-6R+PH-v1 were intravenously administered as single doses of 50 µg/kg for hsIL-6R and 1 mg/kg for antibody and a time profile of total hsIL-6R plasma concentration (A) is shown. Each data point represents the mean ± s.d. (n = 3 each). In the steady-state model, steady-state plasma concentration of approximately 20 ng/mL hsIL-6R was maintained using an infusion pump filled with hsIL-6R solution, and NPH-IgG1, PH-IgG1 and PH-v1 were intravenously administered as single doses of 1 mg/kg and a time profile of total hsIL-6R plasma concentration (B) is shown. Each data point represents the mean ± s.d. (n = 3 each).

In order to evaluate the effect of pH-dependent antigen binding and increased FcRn binding affinity at neutral pH on antigen pharmacokinetics, we used a non-pH-dependent binding antibody against hsIL-6R with hIgG1 constant region (NPH-IgG1) and a pH-dependency-engineered variant (PH-IgG1) (Table S1). hIgG1 has almost no detectable binding to FcRn at pH 7.4, and very weak binding at pH 7.0. In order to increase the binding affinity of PH-IgG1 to either mouse FcRn (mFcRn) or human FcRn (hFcRn) at neutral pH, various hIgG1 Fc variants with mutation(s) in the FcRn binding region (positions 251–258, 286, 288, 307–316, 428 and 433–436 in the EU numbering system) were generated by site-directed mutagenesis. More than 1000 variants were screened by binding to either mFcRn or hFcRn at pH 7.0, and hIgG1 Fc variants for *in vivo* studies were selected (Table 1). The v1 variant was used to evaluate the *in vivo* effect of pH-dependent antigen binding antibody with increased binding affinity to mFcRn at pH 7.0 by administering hsIL-6R to normal mice either on its own or in complex with NPH-IgG1, PH-IgG1 or PH-v1 (Fig. 1A). In this co-injection model, NPH-IgG1 significantly reduced the clearance of hsIL-6R because antigen-antibody complex has lower clearance than the antigen [9], [10]; PH-IgG1 increased the clearance of hsIL-6R to some extent, but was still slower than hsIL-6R alone; while PH-v1 accelerated the clearance of hsIL-6R faster than hsIL-6R alone.

**Table 1:** Mutations and FcRn binding affinity of hIgG1 Fc variants.

Fc variant	K_D_ (nM) at pH7.0		K_D_ (nM) at pH6.0		Mutations
	mouse FcRn	human FcRn	mouse FcRn	human FcRn	
IgG1	3918	88000	237	1377	–
v1	52	NT	3	NT	I332V/N434Y
v2	NT	155	NT	6	M252W/N434W
v3	NT	288	NT	15	M252Y/N434Y
v4	NT	120	NT	8	M252Y/N286E/N434Y
v5	NT	77	NT	5	M252Y/T307Q/Q311A/N434Y
v6	NT	35	NT	3	M252Y/V308P/N434Y
v0	no binding	no binding	no binding	no binding	I253A

Binding affinity (K_D_) of IgG1 and v1 to mFcRn at pH 7.0 and pH 6.0, binding affinity (K_D_) of IgG1, v2-v6 and v0 to hFcRn at pH 7.0 and pH 6.0, and mutations introduced in the Fc region are shown. Mutation sites in the Fc region are described in EU numbering. NT, not tested.

Since this co-injection model may not have reflected the actual therapeutic situation where antibody is exposed to plasma in which steady-state baseline concentration of soluble antigen is present, we evaluated antigen sweeping in a mouse model which maintains steady-state plasma antigen concentration. We administered NPH-IgG1, PH-IgG1 and PH-v1 into normal mice steady-state model (Fig. 1B). Consistent with the co-injection model, NPH-IgG1 significantly increased hsIL-6R plasma concentration; PH-IgG1 reduced that increase but an increase over the baseline was still observed; and PH-v1 actively eliminated hsIL-6R from the plasma and reduced the plasma hsIL-6R concentration approximately 150-fold below the baseline, demonstrating that engineered antibody with pH-dependent binding antibody and increased binding affinity to FcRn at neutral pH can eliminate the antigen form plasma *in vivo*.

### Effect of Antigen Sweeping by Sweeping Antibody on Antigen Antagonism *in vivo*

**Figure 2:**
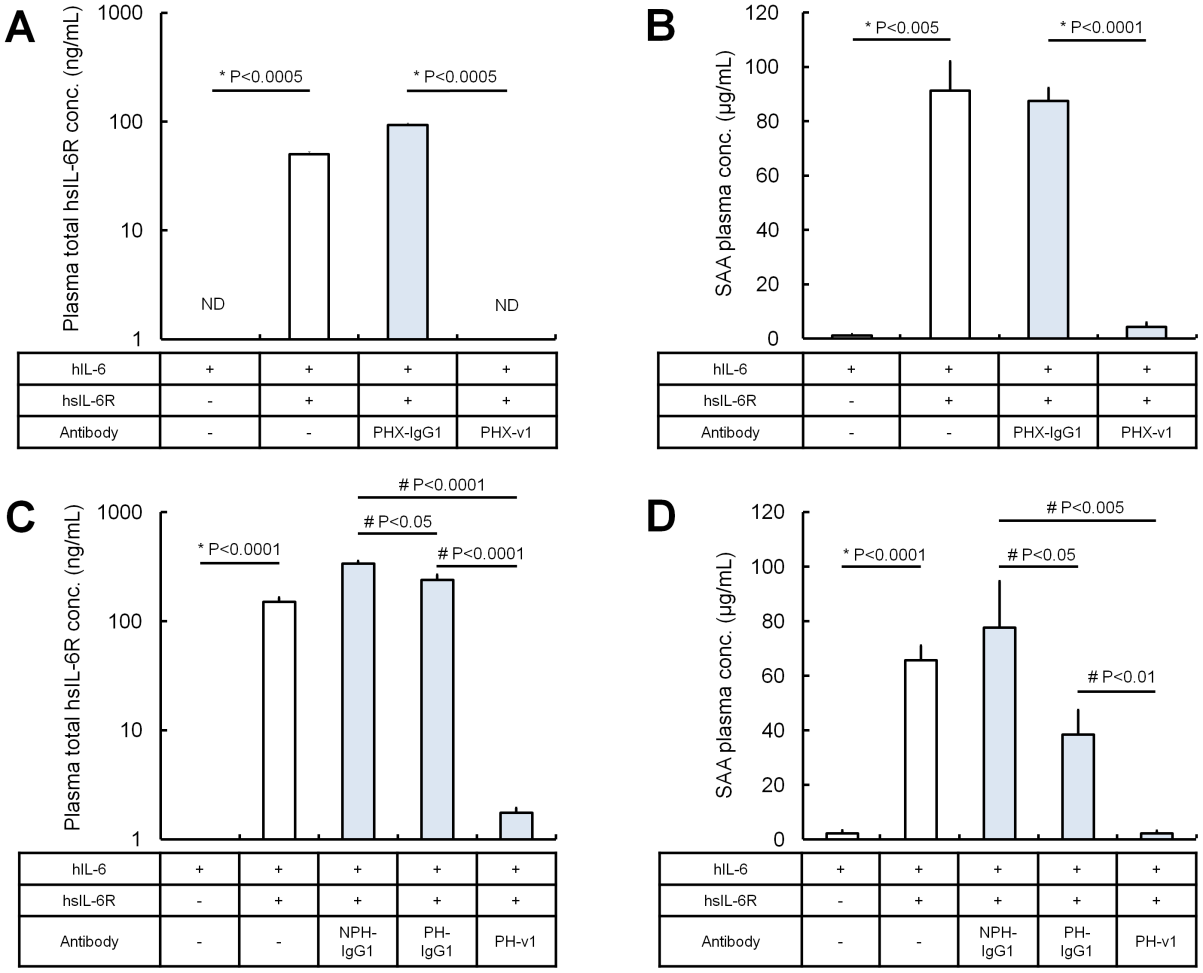
*In vivo* study of sweeping antibodies in a normal mice hsIL-6R trans-signaling model. Effect of antibodies on the total hsIL-6R plasma concentration and SAA plasma concentration (as a marker for hsIL-6R antagonism) were evaluated. hsIL-6R was intravenously administered as a single dose of 250 µg/kg. At 2 h, non-neutralizing antibodies PHX-IgG1 and PHX-v1 were intravenously administered as single doses of 30 mg/kg (A, B), and neutralizing antibodies NPH-IgG1, PH-IgG1 and PH-v1 were intravenously administered as single doses of 0.03 mg/kg (C, D). At 24 h, hIL-6 was intravenously administered as a single dose of 8 µg/kg. Total hsIL-6R plasma concentration (A, C) and SAA plasma concentration (B, D) at 30 h is shown. Each data represents the mean ± s.d. for total hsIL-6R plasma concentration and the mean ± s.e. for SAA plasma concentration (n = 3–7 each). ND, not detected (below 0.195 ng/mL). Statistical significance was determined by t-test (*) or Tukey’s multiple comparison test (#) for total hsIL-6R and SAA plasma concentration.

To evaluate the effect of antigen sweeping by sweeping antibody on antigen antagonism *in vivo*, *in vivo* efficacy of the anti-hsIL-6R sweeping antibodies was tested in a normal mouse hsIL-6R trans-signaling model [31], which exhibits an increase in SAA dependent on hIL-6/hsIL-6R-mediated trans-signaling. We generated PHX-IgG1, a pH-dependent binding antibody against hsIL-6R without hsIL-6R neutralizing activity *in vitro* (Table S1), and its Fc variant PHX-v1 with increased binding affinity to mFcRn at neutral pH. In the first study, PHX-IgG1 and PHX-v1 were administered at antibody dosage of 30 mg/kg, and plasma concentration of hsIL-6R and SAA, as a pharmacodynamic marker of hsIL-6R antagonism, are shown (Fig. 2A,B). While PHX-IgG1 could not inhibit SAA production at all, PHX-v1 significantly inhibited SAA production *in vivo* by directly sweeping hsIL-6R from the plasma, despite having no neutralizing activity *in vitro*. In the next study, antibodies with hsIL-6R neutralizing activity, NPH-IgG1, PH-IgG1 and PH-v1, were administered at antibody dosage of 0.03 mg/kg. While NPH-IgG1 and PH-IgG1 with hsIL-6R neutralizing activity *in vitro* could not completely inhibit SAA production at this dosage, PH-v1, with both neutralizing and sweeping activity, completely inhibited SAA production (Fig. 2C,D).

### Antigen Sweeping Requires Both pH-Dependent Antigen Binding and Increased FcRn Binding Affinity at Neutral pH

**Figure 3:**
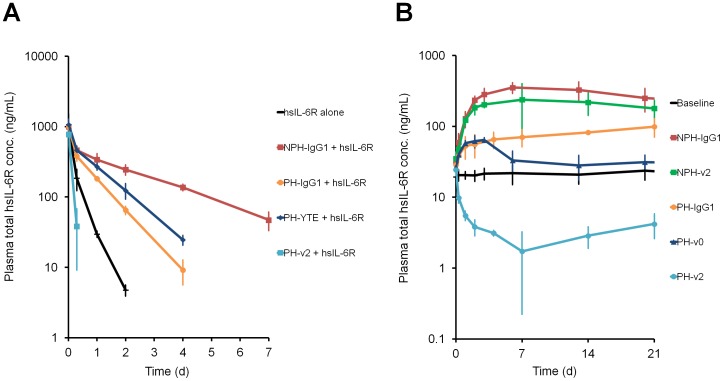
Characterization of sweeping antibody in hFcRn-Tgm. (A) *In vivo* study of NPH-IgG1, PH-IgG1, PH-YTE and PH-v2 in hFcRn-Tgm. Effect of antibodies on the total hsIL-6R plasma concentration was evaluated in a co-injection model. hsIL-6R, hsIL-6R+NPH-IgG1, hsIL-6R+PH-IgG1, hsIL-6R+PH-YTE and hsIL-6R+PH-v2 were intravenously administered as single doses of 50 µg/kg for hsIL-6R and 1 mg/kg for antibody and a time profile of total hsIL-6R plasma concentration is shown. Each data point represents the mean ± s.d. (n = 3 each). (B) Effect of pH-dependent antigen binding and increased binding affinity to FcRn at neutral pH on antigen sweeping in hFcRn-Tgm steady-state model with hsIL-6R plasma concentration of approximately 20 ng/mL. NPH-IgG1, NPH-v2, PH-IgG1, PH-v2 and PH-v0 were intravenously administered as single doses of 1 mg/kg. Time profile of total hsIL-6R plasma concentration is shown. Each data point represents the mean ± s.d. (n = 3 each).

For clinical application of sweeping antibody, further studies of antigen sweeping were conducted using an hFcRn system. The v2 variant with increased binding affinity to hFcRn at neutral pH was generated (Table 1). As a control for v2 variant, a YTE variant previously reported as improving the half-life [19] with increased binding affinity to hFcRn at acidic pH but not significantly at neutral pH, was used. In a co-injection model, hsIL-6R was administered to hFcRn-Tgm either on its own or in complex with NPH-IgG1, PH-IgG1, PH-YTE and PH-v2 (Fig. 3A). Consistent with the study using normal mice, PH-v2 markedly accelerated the clearance of hsIL-6R faster than hsIL-6R alone in hFcRn-Tgm. On the other hand, PH-YTE exerted slightly slower clearance of hsIL-6R than PH-IgG1.

To further clarify the molecular requirement to achieve antigen sweeping, we administered NPH-IgG1, PH-IgG1, NPH-v2, PH-v2 and PH-v0 to hFcRn-Tgm steady-state model (Fig. 3B). NPH-v2, a non-pH-dependent binding antibody with increased binding affinity to hFcRn at neutral pH, increased hsIL-6R plasma concentration above the baseline to a similar level to NPH-IgG1; PH-v0, a pH-dependent binding antibody with no hFcRn binding [32], also increased hsIL-6R plasma concentration to a similar level to PH-IgG1, but only transiently; and only PH-v2, a pH-dependent binding antibody with increased binding affinity to hFcRn at neutral pH, actively eliminated hsIL-6R from the plasma. This clearly demonstrates that both pH-dependent antigen binding and increased binding affinity to FcRn at neutral pH are required for antigen sweeping.

### Effect of Endogenous IgG Competition on Antigen Sweeping

Because mouse IgG does not bind to hFcRn [33], hFcRn-Tgm has substantially no endogenous IgG competing with sweeping antibody for hFcRn, which might not reflect the clinical situation in which there is high endogenous human IgG (hIgG) concentration [20]. To evaluate the effect of endogenous IgG on antigen sweeping, NPH-IgG1, PH-IgG1 and PH-v2 alone or together with 1 g/kg of hIgG, which mimics endogenous IgG, were administered to hFcRn-Tgm steady-state model. hsIL-6R sweeping by PH-v2 was attenuated when hIgG as endogenous IgG was present (Fig. S1).

### Effect of hFcRn Binding Affinity at Neutral pH on Antigen Sweeping in Human FcRn Transgenic Mice

Since FcRn binding at neutral pH is required for antigen sweeping, it is assumed that binding affinity (K_D_) to FcRn at neutral pH would affect the antigen sweeping profile. In addition, previous studies have shown that increasing FcRn binding affinity at neutral pH either increased or did not affect the antibody clearance [21]–[24]. In order to assess the effect of FcRn binding affinity at neutral pH on antigen sweeping and antibody pharmacokinetics, Fc variants (v3-v6) with various binding affinity to hFcRn at pH 7.0 were generated (Table 1).

**Figure 4:**
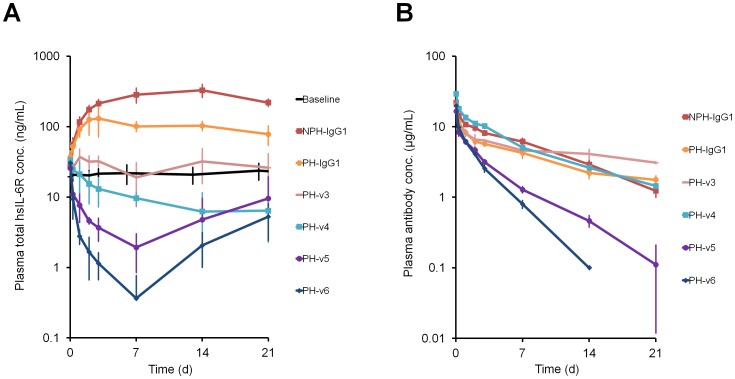
Effect of hFcRn binding affinity at neutral pH on antigen sweeping profile in hFcRn-Tgm. (A, B) Effect of FcRn binding affinity at pH 7.0 on antigen sweeping and antibody pharmacokinetics in hFcRn-Tgm steady-state model with hsIL-6R concentration of approximately 20 ng/mL in the presence of human IgG. NPH-IgG1, PH-IgG1, PH-v3, v4, v5 and v6 were intravenously administered as single doses of 1 mg/kg with 1 g/kg of hIgG. Time profiles of total hsIL-6R plasma concentration (A) and antibody plasma concentration (B) are shown. Each data point represents the mean ± s.d. (n = 3–6 each).

Antigen sweeping and antibody pharmacokinetics of NPH-IgG1, PH-IgG1 and its Fc variants were evaluated in hFcRn-Tgm steady-state model in the presence of hIgG (Fig. 4A,B, Table S2). Compared to IgG1, the v3 variant, with K_D_ 288 nM at pH 7.0, slightly prolonged the antibody pharmacokinetics; moreover, PH-v3 reduced total hsIL-6R plasma concentration to a similar level as baseline concentration. Notably, the v4 variant, with K_D_ 120 nM at pH 7.0, reduced total hsIL-6R plasma concentration below the baseline level while the antibody pharmacokinetics was maintained. Total hsIL-6R plasma concentration of PH-v4 was 50-fold lower than NPH-IgG1 while the antibody plasma concentration was comparable. The study using the v5 and v6 variants with, respectively, K_D_ 77 and 35 nM at pH 7.0 has demonstrated that variants with stronger hFcRn binding affinity exhibited more extensive antigen sweeping and lower minimum total hsIL-6R plasma concentration, but had increased antibody clearance and a shorter duration of antigen sweeping (faster recovery to baseline). Specifically, the v6 variant reduced total hsIL-6R plasma concentration approximately 1000-fold compared to NPH-IgG1, while the antibody clearance was increased only by 4-fold.

### Sweeping Antibody Antagonizes High Concentration Antigen where Conventional Antibody is Ineffective

**Figure 5:**
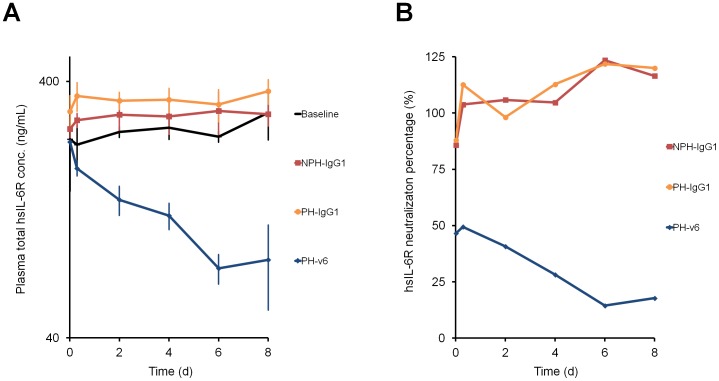
Effect of sweeping antibody on high plasma concentration antigen. Effect of NPH-IgG1, PH-IgG1 and PH-v6 on a hFcRn-Tgm steady-state model with high hsIL-6R concentration of approximately 250 ng/mL in the presence of human IgG. NPH-IgG1, PH-IgG1 and PH-v6 were intravenously administered as multiple doses of 0.01 mg/kg every other day. Molar baseline hsIL-6R concentration (6.6 nM) is 5-fold higher than antibody concentration at 15 min (1.3 nM). Time profiles of total hsIL-6R plasma concentration (A) and free hsIL-6R percentage over control (B) are shown. Each data point represents the mean ± s.d. for total hsIL-6R concentration (n = 3–5 each). Free hsIL-6R percentage over control is determined from the pooled plasma sample of n = 3–5 each.

The v6-type sweeping antibody with hFcRn binding affinity of 35 nM provided short-lasting but extensive 1000-fold reduction of antigen plasma concentration compared to conventional antibody. To understand its therapeutic advantage, NPH-IgG1, PH-IgG1 and PH-v6 at doses of 0.01 mg/kg were administered to hFcRn-Tgm steady-state model with high plasma hsIL-6R concentration (250 ng/mL) every other day in the presence of hIgG (Fig. 5 A, B). Multiple dosing of NPH-IgG1 and PH-IgG1 achieved no hsIL-6R neutralization throughout the study because molar hsIL-6R concentration was higher than that of antibody (molar antibody concentration was approximately 5-fold lower than the total antigen concentration immediately after the first administration); however, PH-v6 gradually reduced the total hsIL-6R plasma concentration, enabling a neutralization of hsIL-6R at Day 8.

## Discussion

In this study, we have demonstrated that simultaneous engineering of pH-dependent antigen binding and increased FcRn binding affinity at neutral pH actively eliminated the antigen from the plasma, creating “sweeping antibody”. Importantly, both pH-dependent antigen binding and increased FcRn binding affinity at neutral pH was required for antigen sweeping, mimicking the function of the ligand-sweeping endocytic receptors that we previously mentioned.

When targeting soluble antigen with monoclonal antibody, conventional antibody (NPH-IgG1) remains bound to the soluble antigen within the acidic endosome (Fig. S2A) and thereby inhibits the antigen degradation by lysosome, resulting in accumulation of the antigen in the plasma. We have recently reported that engineered antibody with pH-dependent antigen binding (PH-IgG1), named recycling antibody, dissociates the soluble antigen in the acidic endosome and the dissociated antigen is then transferred to lysosome and degraded (Fig. S2B) [16]. However, our current study demonstrates that pH-dependent antigen binding alone could not actively eliminate the antigen from plasma. This is because intact IgG1 does not bind to FcRn on the cell surface at neutral pH [25], and the antibody-antigen complex is only marginally taken up into the cell by pinocytosis, which limits the rate of antigen degradation.

Previous studies have demonstrated that Fc-engineering to increase the binding affinity to FcRn at acidic pH improved the endosomal recycling efficiency and prolonged the pharmacokinetics of the antibody [20], [25], [26]. However, a simultaneous increase of binding affinity at neutral pH did not prolong [21], [22], or even shortened [23], [24], the pharmacokinetics because of inefficient antibody release from FcRn back to plasma after transporting it back to the cell surface, providing no therapeutic merit. Increasing FcRn binding affinity at neutral pH would anchor the antibody to the cell surface, similarly to an endocytic receptor, and enhance cellular uptake of antibody-antigen complex by FcRn-mediated endocytosis. However, our study demonstrated that antibody with increased FcRn binding affinity at neutral pH without pH-dependent antigen binding (NPH-v2) could not actively eliminate the antigen from plasma because the antigen is also recycled back to plasma by FcRn in the antibody-antigen complex.

Combining a pH-dependent antigen binding with increased FcRn binding at only acidic pH (PH-YTE) attenuated rather than accelerated antigen clearance compared to pH-dependent antigen-binding antibody with wild type IgG1 (PH-IgG1), probably because the improved endosomal recycling efficiency [19] also applied to the antigen that remained bound to the antibody. In addition, non-FcRn binding Fc (PH-NB) could not actively eliminate the antigen from plasma because, similar to PH-IgG1, the uptake of antibody-antigen complex into the cell was marginal.

Antigen sweeping was only achieved by the combination of pH-dependent antigen binding with increased FcRn binding affinity at neutral pH (PH-v2). These studies support the following mechanism of sweeping antibody, mimicking the process of rapid ligand sweeping by endocytic receptors [13]–[15] (Fig. S2C): (i) increasing binding affinity to FcRn at neutral pH anchors the antibody to the cell surface and provides FcRn-mediated cellular uptake of antibody-antigen complex, (ii) pH-dependent dissociation of antibody-antigen complex enables selective degradation of the antigen, (iii) FcRn-mediated recycling of the free antibody to the cell surface enables another round of the cycle. Because this cycle has a rapid turnover rate and FcRn is broadly expressed in the body, sweeping antibody can effectively eliminate the antigen from plasma.

As our results show, this antigen-sweeping activity can be successfully applied either to antibody which has no *in vitro* hsIL-6R neutralizing activity (PHX-v1) to create *in vivo* inhibition of hsIL-6R/hIL-6-mediated trans-signaling or to convert conventional antibody with *in vitro* activity (NPH-IgG1) into sweeping antibody (PH-v1) to further potentiate the *in vivo* signaling inhibition. This study demonstrated that *in vivo* efficacy of sweeping antibody required no *in vitro* biological activity, indicating that sweeping antibody could antagonize multi-epitope antigen (antigen with multiple functional epitope) or toxic antigen with no functional epitope, which cannot be antagonized with a conventional antibody, by directly eliminating the antigen from plasma. Moreover, this study also demonstrated that conventional antibody with biological activity *in vitro* can be further potentiated *in vivo* by engineering the antibody into sweeping antibody, indicating that engineering conventional antibody into sweeping antibody could be an alternative approach to enhancing the therapeutic efficacy of conventional antibody.

Since sweeping antibody would compete with endogenous IgG to bind to FcRn, it was assumed that endogenous IgG would affect the efficiency of antigen sweeping. A similar phenomenon has been reported for antibody-dependent cellular cytotoxicity mediated by Fc gamma receptor binding, which was significantly inhibited by the presence of endogenous hIgG [34]. As expected, antigen sweeping in hFcRn-Tgm was significantly attenuated in the presence of hIgG when hIgG concentration was maintained at an average of 10 mg/mL (reflecting the clinical situation where endogenous hIgG is approximately 10 mg/mL [20]). This demonstrates that endogenous IgG is an important factor in the efficacy of sweeping antibody when considering clinical applications. It has been reported that IgG with increased FcRn binding at neutral pH (Abdeg) accelerates the clearance of endogenous IgG by blocking FcRn [35]. However, we did not observe accelerated clearance of hIgG in the presence of v4-type sweeping antibody (data not shown). Since reported Abdeg (with FcRn binding affinity of 7.4 nM at pH 7.2) accelerated the clearance of endogenous IgG at a dose of approximately 8 mg/kg, it is expected that v4-type sweeping antibody (with FcRn binding affinity of 120 nM at pH 7.0 significantly lower than Abdeg) would not accelerate the clearance of endogenous IgG at a therapeutically relevant dosage, although a high dosage of v6-type sweeping antibody (with stronger FcRn binding affinity of 35 nM at pH 7.0) may have some effect on the clearance of endogenous IgG.

The effect of hFcRn binding affinity at neutral pH on the antigen sweeping profile and antibody pharmacokinetics was investigated by evaluating Fc variants v3 to v6 in hFcRn-Tgm in the presence of hIgG. The results clearly demonstrate that both antigen sweeping and antibody pharmacokinetics depend on hFcRn binding affinity at neutral pH. By increasing the binding affinity at neutral pH, the extent of antigen sweeping (reflected by minimum total hsIL-6R plasma concentration (Table S2)) was enhanced but the duration of antigen sweeping was shortened and antibody clearance was increased. Compared to conventional antibody (NPH-IgG1), all sweeping antibody exhibited stronger reduction of free antigen plasma concentration, which determines the *in vivo* efficacy as a therapeutic antibody, and the extent and the duration of free antigen reduction depended on hFcRn binding affinity (Fig. S3).

Sweeping antibody with moderate hFcRn binding affinity at neutral pH provides moderate but long-acting antigen sweeping. Specifically, compared to conventional antibody (NPH-IgG1), sweeping antibody with hFcRn binding affinity of 120 nM (PH-v4) maintains a similar antibody plasma concentration and provides long-lasting approximately 50-fold reduction of total antigen plasma concentration. Importantly, this demonstrates that the antigen, not the antibody, is selectively eliminated from the plasma. To systematically understand the therapeutic advantage of this v4-type sweeping antibody, modeling and simulation [30] was conducted based on the experimental result of the hFcRn-Tgm study (Fig. S4A, Table S3). The simulation was conducted to calculate the dosage required to neutralize 95% of hsIL-6R (baseline 250 ng/mL) by once-a-month dosing using conventional, pH-dependent antigen binding, and v4-type sweeping antibodies with different binding affinity to hsIL-6R (Fig. S4B). In the simulation study, the dosage of conventional antibody cannot be lowered below 45 mg/kg even with infinite affinity, whereas sweeping antibody with only 0.1 nM affinity can be effective at 1.4 mg/kg. This demonstrates that v4-type sweeping antibody provides more than 30-fold reduction of dosage over conventional antibody even with infinite affinity, a level which can never be achieved with conventional antibody.

On the other hand, sweeping antibody with hFcRn binding affinity below 80 nM provides short-lasting but extensive reduction of antigen plasma concentration compared to conventional antibody. Specifically, sweeping antibody with hFcRn binding affinity of 35 nM (PH-v6) reduces antigen concentration approximately 1000-fold compared to conventional antibody, while the antibody clearance is increased only 4-fold. To understand the therapeutic advantage of v6-type sweeping antibody, antibodies were tested under conditions in which an excess molar amount of antigen was present in plasma, mimicking the therapeutic situation where antigen is present at a high concentration. This excess amount of antigen, which, as expected, conventional antibody (NPH-IgG1) or pH-dependent binding antibody (PH-IgG1) could not antagonize, was antagonized by sweeping antibody (PH-v6) by reducing the plasma antigen concentration below the baseline. This study demonstrated that sweeping antibody could antagonize high concentration antigen against which conventional or pH-dependent antigen binding antibody, even with infinite affinity, would be completely ineffective.

We believe that sweeping antibody, an engineered monoclonal antibody with novel antigen-sweeping activity, provides potential advantages over conventional antibody that can only bind to the antigen and accumulates the antigen in plasma. First, sweeping antibody could be applied to high concentration antigens or antigens with rapid clearance which conventional antibodies, even with infinite affinity, have previously had difficulty in targeting. Second, by directly eliminating the antigen from plasma, sweeping antibody could be applied to antagonize multi-epitope antigen or toxic antigens without functional epitope, which cannot be simply antagonized by a conventional antibody. These two points suggest that sweeping antibody may expand the target antigen space of therapeutic monoclonal antibody to include target antigens which were previously undruggable by conventional monoclonal antibody. Third, sweeping antibody could provide an alternative approach to affinity maturation against the antigen by reducing the plasma antigen concentration to potentiate the efficacy of conventional antibody [36]. Fourth, sweeping antibody could provide a significant advantage over conventional antibody (even assuming infinite affinity) in dosing by enabling the convenience of subcutaneous and less frequent injections, or in manufacturing by reducing the cost. Since changing the binding affinity to hFcRn generates antibodies with different extent and duration of antigen sweeping, antigen-sweeping profiles can be readily customized. We have applied sweeping antibody technology to various antigens such as IL6, IgA, soluble plexin A1, soluble CD4 and other antigens. We have identified pH-dependent antibodies against each of these antigens and engineered them to bind to FcRn at neutral pH. All of these antibodies demonstrated similar antigen sweeping effect that is shown in this study using hsIL-6R (data not shown). These results suggest that sweeping antibody can be broadly applicable to various antigens.

## Supporting Information

Figure S1 **Effect of high concentration hIgG on antigen sweeping in hFcRn-Tgm.** NPH-IgG1, PH-IgG1 and PH-v2 were intravenously administered as single doses of 1 mg/kg either with or without 1 g/kg of hIgG to hFcRn-Tgm with steady-state hsIL-6R concentration of approximately 20 ng/mL. Time profile of total hsIL-6R plasma concentration is shown. Each data point represents the mean ± s.d. (n = 3 each).
(TIF)

Figure S2 **Proposed mode of action of sweeping antibody in comparison with conventional and pH-dependent binding antibody.** (A) Conventional antibody bound to soluble antigen is non-specifically taken up by pinocytosis, and binds to FcRn in acidic endosome. Antibody-antigen complex is recycled back to the cell surface and released from FcRn back to plasma. (B) pH-dependent binding antibody (recycling antibody) bound to soluble antigen is non-specifically taken up by pinocytosis, and binds to FcRn in acidic endosome, while antigen is dissociated from the antibody, transferred into lysosome and degraded. Antibody is recycled back to the cell surface by FcRn, released from FcRn back to plasma and binds to another antigen, allowing single antibody to bind to antigen multiple times. (C) Sweeping antibody bound to soluble antigen is rapidly taken up by FcRn-mediated endocytosis. In acidic endosome, antibody binds to FcRn, and antigen is dissociated from the antibody, transferred into lysosome and degraded. Antibody is recycled back to the cell surface and either released from FcRn back to plasma or stays bound to FcRn on the cell surface to bind to another antigen. Rapid FcRn-mediated uptake allows enhanced lysosomal antigen degradation rate.
(TIF)

Figure S3 **Antigen sweeping profile of antibodies with different hFcRn binding affinity at neutral pH in hFcRn-Tgm.** Effect of FcRn binding affinity at pH 7.0 on antigen sweeping and antibody pharmacokinetics in hFcRn-Tgm with steady-state hsIL-6R concentration of approximately 20 ng/mL in the presence of human IgG. NPH-IgG1, PH-IgG1, PH-v3, v4, v5 and v6 were intravenously administered as single doses of 1 mg/kg with 1 g/kg of hIgG. Theoretical free hsIL-6R plasma concentration was calculated from plasma antibody concentration, total hsIL-6R concentration and binding affinity to hsIL-6R. Time profile of theoretical free hsIL-6R plasma concentration is shown.
(TIF)

Figure S4 **Modeling and simulation of sweeping antibody.** (A) Antibody-antigen dynamic model of sweeping antibody. Antibody is injected intravenously to the central compartment and distributed to the peripheral compartment. Antibody binds to the antigen in the central compartment. Antibody, antigen and antibody-antigen complex are eliminated from the central compartment. Effect of pH-dependent binding and increased binding affinity to FcRn is reflected in the elimination rate of antibody-antigen complex. Parameters used in this model are k_synthe_ (rate constant of antigen synthesis), C_antigen,baseline_ (baseline concentration of antigen), k_el,antigen_ (elimination rate constant of antigen), Vd1_antigen_ (volume of distribution of antigen), k_12,antigen_ (transfer rate constant of antigen from central to peripheral compartment), k_21,antigen_ (transfer rate constant of antigen from peripheral to central compartment), k_el,mab_ (elimination rate constant of antibody), Vd1_mab_ (volume of distribution of antibody), k_12,mab_ (transfer rate constant of antibody from central to peripheral compartment), k_21,mab_ (transfer rate constant of antibody from peripheral to central compartment) and k_el,complex_ (elimination rate constant of antigen in complex with antibody). Note that antibody in complex with antigen is eliminated at the rate of k_el,mab_. (B) Simulation of required dosage to neutralize antigen (baseline concentration 250 ng/mL) by 95% at trough with dosing once a month using conventional antibody (non-pH dependent binding IgG1 antibody), pH-dependent binding IgG1 antibody and v4-type sweeping antibody with different binding affinity to the antigen. Relationship between the antigen binding affinity (K_D_) and the antibody dosage required to achieve once monthly dosing is shown.
(TIF)

Table S1 **Binding affinity of the anti-IL-6R IgG1 antibodies.**
(TIF)

Table S2 **Antibody clearance and minimum or maximum total hsIL-6R plasma concentration for tested antibodies.**
(TIF)

Table S3 **Fitted pharmacokinetic parameters in antibody-antigen dynamic model.**
(TIF)
