# Supplementary figures and images for: Engineered Monoclonal Antibody with Novel Antigen-Sweeping Activity In Vivo
Source: PLoS One. 2013 May 7;8(5):e63236. doi: 10.1371/journal.pone.0063236 (PMC3646756; doi:10.1371/journal.pone.0063236)

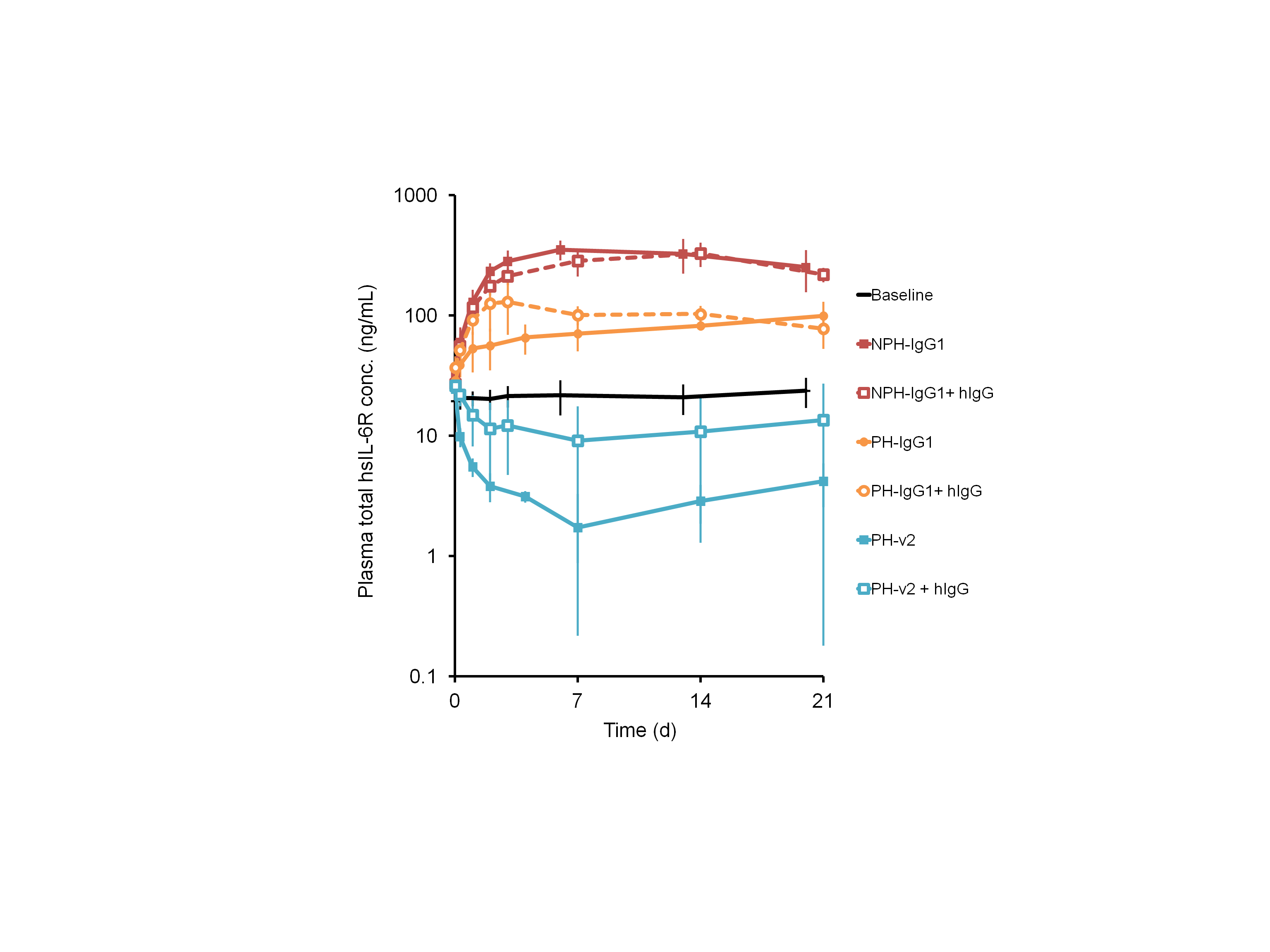

Supplement: Figure S1 — Effect of high concentration hIgG on antigen sweeping in hFcRn-Tgm. NPH-IgG1, PH-IgG1 and PH-v2 were intravenously administered as single doses of 1 mg/kg either with or without 1 g/kg of hIgG to hFcRn-Tgm with steady-state hsIL-6R concentration of approximately 20 ng/mL. Time profile of total hsIL-6R plasma concentration is shown. Each data point represents the mean ± s.d. (n = 3 each). (TIF) [file pone.0063236.s001.tif]

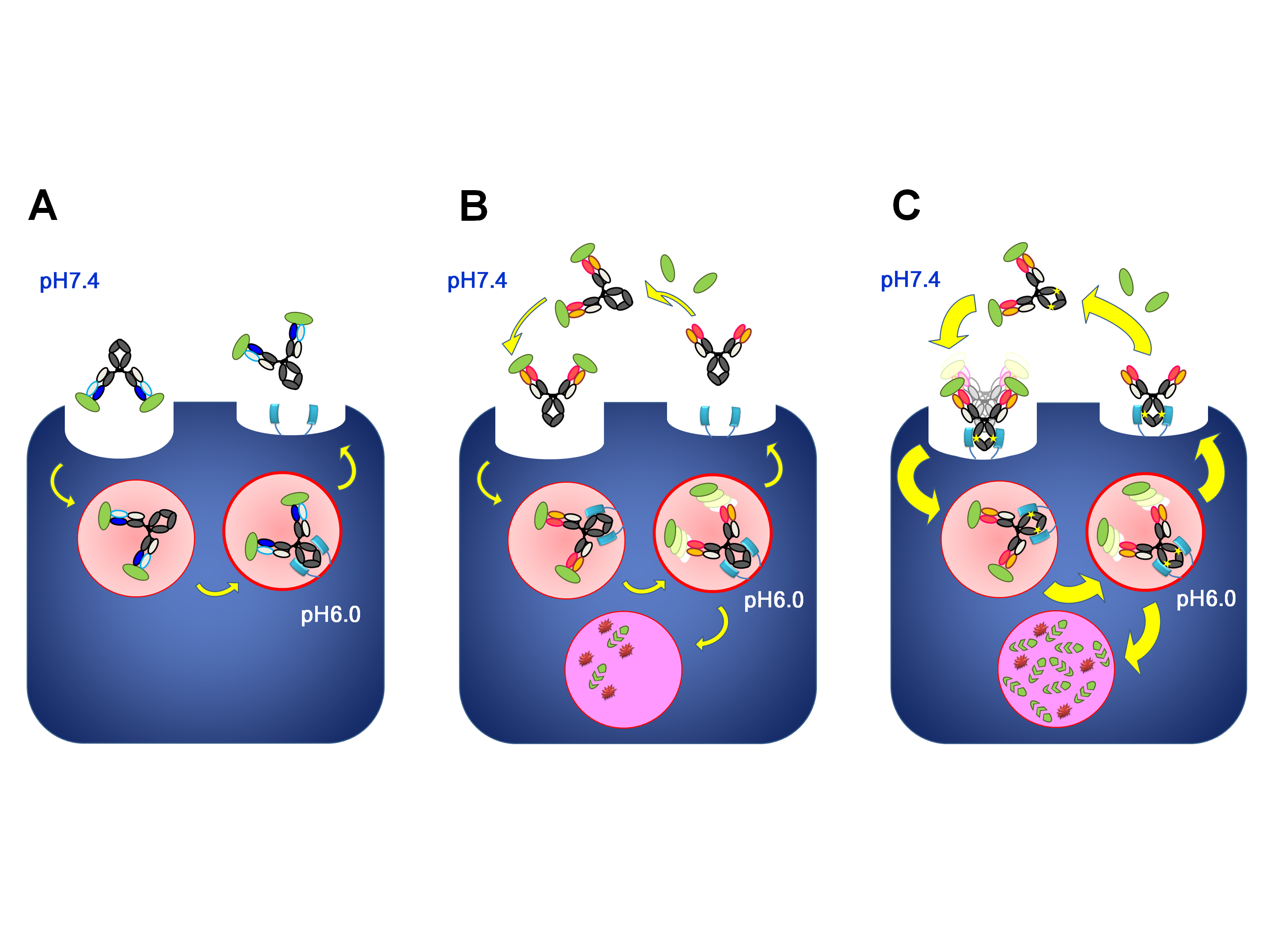

Supplement: Figure S2 — Proposed mode of action of sweeping antibody in comparison with conventional and pH-dependent binding antibody. (A) Conventional antibody bound to soluble antigen is non-specifically taken up by pinocytosis, and binds to FcRn in acidic endosome. Antibody-antigen complex is recycled back to the cell surface and released from FcRn back to plasma. (B) pH-dependent binding antibody (recycling antibody) bound to soluble antigen is non-specifically taken up by pinocytosis, and binds to FcRn in acidic endosome, while antigen is dissociated from the antibody, transferred into lysosome and degraded. Antibody is recycled back to the cell surface by FcRn, released from FcRn back to plasma and binds to another antigen, allowing single antibody to bind to antigen multiple times. (C) Sweeping antibody bound to soluble antigen is rapidly taken up by FcRn-mediated endocytosis. In acidic endosome, antibody binds to FcRn, and antigen is dissociated from the antibody, transferred into lysosome and degraded. Antibody is recycled back to the cell surface and either released from FcRn back to plasma or stays bound to FcRn on the cell surface to bind to another antigen. Rapid FcRn-mediated uptake allows enhanced lysosomal antigen degradation rate. (TIF) [file pone.0063236.s002.tif]

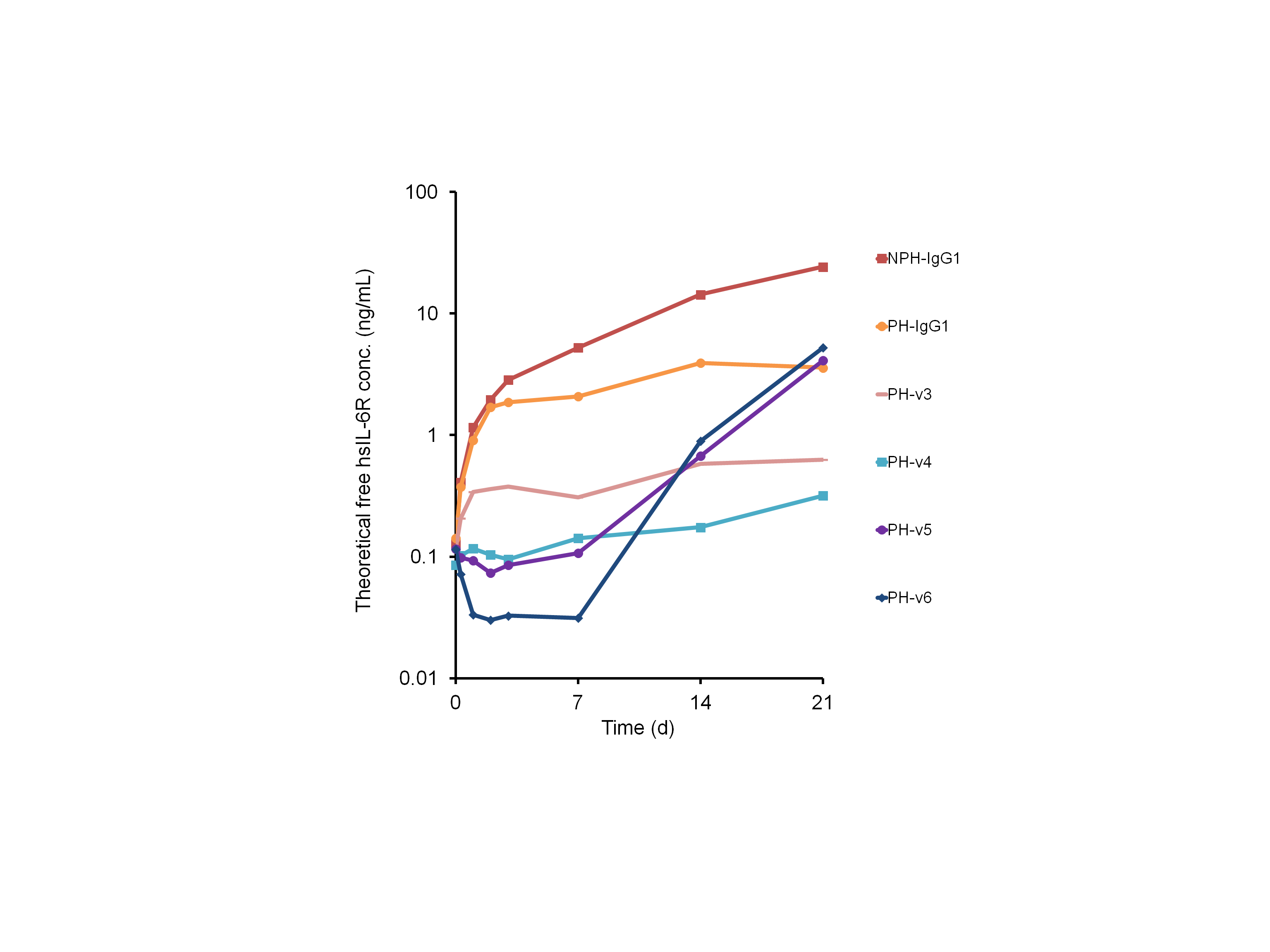

Supplement: Figure S3 — Antigen sweeping profile of antibodies with different hFcRn binding affinity at neutral pH in hFcRn-Tgm. Effect of FcRn binding affinity at pH 7.0 on antigen sweeping and antibody pharmacokinetics in hFcRn-Tgm with steady-state hsIL-6R concentration of approximately 20 ng/mL in the presence of human IgG. NPH-IgG1, PH-IgG1, PH-v3, v4, v5 and v6 were intravenously administered as single doses of 1 mg/kg with 1 g/kg of hIgG. Theoretical free hsIL-6R plasma concentration was calculated from plasma antibody concentration, total hsIL-6R concentration and binding affinity to hsIL-6R. Time profile of theoretical free hsIL-6R plasma concentration is shown. (TIF) [file pone.0063236.s003.tif]

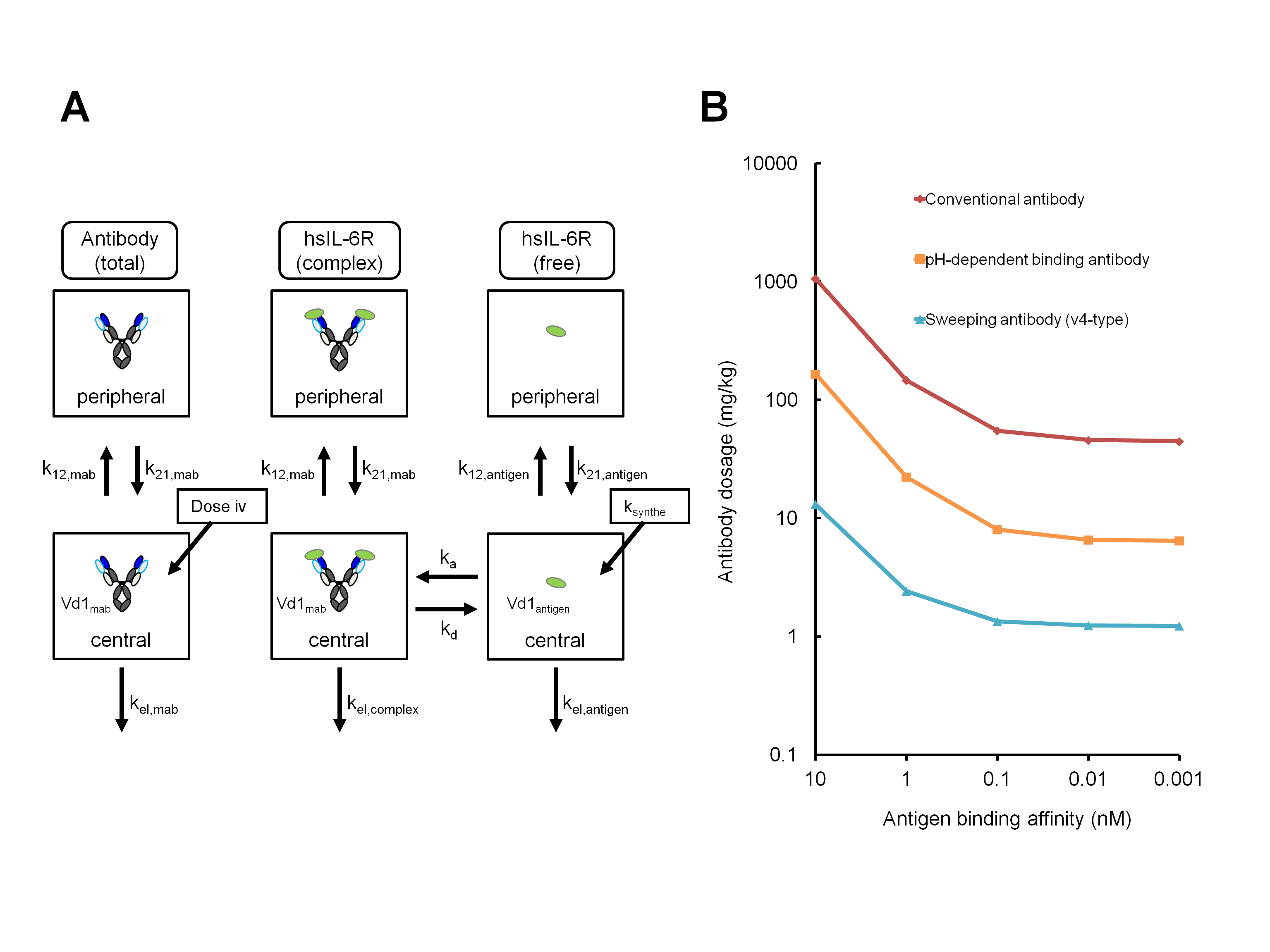

Supplement: Figure S4 — Modeling and simulation of sweeping antibody. (A) Antibody-antigen dynamic model of sweeping antibody. Antibody is injected intravenously to the central compartment and distributed to the peripheral compartment. Antibody binds to the antigen in the central compartment. Antibody, antigen and antibody-antigen complex are eliminated from the central compartment. Effect of pH-dependent binding and increased binding affinity to FcRn is reflected in the elimination rate of antibody-antigen complex. Parameters used in this model are ksynthe (rate constant of antigen synthesis), Cantigen,baseline (baseline concentration of antigen), kel,antigen (elimination rate constant of antigen), Vd1antigen (volume of distribution of antigen), k12,antigen (transfer rate constant of antigen from central to peripheral compartment), k21,antigen (transfer rate constant of antigen from peripheral to central compartment), kel,mab (elimination rate constant of antibody), Vd1mab (volume of distribution of antibody), k12,mab (transfer rate constant of antibody from central to peripheral compartment), k21,mab (transfer rate constant of antibody from peripheral to central compartment) and kel,complex (elimination rate constant of antigen in complex with antibody). Note that antibody in complex with antigen is eliminated at the rate of kel,mab. (B) Simulation of required dosage to neutralize antigen (baseline concentration 250 ng/mL) by 95% at trough with dosing once a month using conventional antibody (non-pH dependent binding IgG1 antibody), pH-dependent binding IgG1 antibody and v4-type sweeping antibody with different binding affinity to the antigen. Relationship between the antigen binding affinity (KD) and the antibody dosage required to achieve once monthly dosing is shown. (TIF) [file pone.0063236.s004.tif]
